# Epidemiology of Bovine Pestiviruses Circulating in Italy

**DOI:** 10.3389/fvets.2021.669942

**Published:** 2021-06-02

**Authors:** Camilla Luzzago, Nicola Decaro

**Affiliations:** ^1^Department of Veterinary Medicine, Coordinated Research Center “EpiSoMI”, University of Milano, Milano, Italy; ^2^Department of Veterinary Medicine, University of Bari Aldo Moro, Valenzano, Italy

**Keywords:** Italy, cattle, Bovine viral diarrhea virus 1, Bovine viral diarrhea virus 2, HoBi-like pestivirus, epidemiology, genetic diversity

## Abstract

Pestiviruses are widespread and economically important pathogens of cattle and other animals. *Pestivirus A* (formerly known as Bovine viral diarrhea virus 1, BVDV-1), *Pestivirus B* (Bovine viral diarrhea virus 2, BVDV-2), and *Pestivirus H* (HoBi-like pestivirus, HoBiPeV) species are infecting primarily cattle. Like other RNA viruses, pestiviruses are characterized by a high degree of genetic variability. This high rate of variability is revealed by the existence of a number of viral subgenotypes within each species. In cattle, the highest number of pestivirus subgenotypes has been documented in European countries, particularly in Italy. The aim of this review is to report an up-to-date overview about the genetic diversity of pestiviruses in Italian cattle herds. All three bovine pestiviruses species have been identified in cattle population with variable frequency and geographical distribution. The genetic diversity of Italian pestiviral strains may have diagnostic and immunological implications, affecting the performance of diagnostic tools and the full cross-protection elicited by commercially available vaccines. Implementation and strengthening of coordinated approaches for bovine pestivirus control in Italy are recommended. Therefore, it would be extremely important to increase control and restriction measures to the trade of cattle and biological products of bovine origin, including those containing fetal bovine serum.

## Introduction

Pestiviruses are widespread and economically important pathogens of cattle (1). Pestivirus infections are associated with a wide range of clinical forms, including subclinical form, gastroenteritis, reproductive failures, and hemorrhagic systemic disease, and with profound immunosuppression that increases the susceptibility of infected cattle to secondary infections (2–4).

Genus *Pestivirus* in the family of *Flaviviridae* is composed of 11 recognized species, *Pestivirus A* (formerly known as Bovine viral diarrhea virus 1, BVDV-1), *Pestivirus B* (Bovine viral diarrhea virus 2, BVDV-2), *Pestivirus C* (Classical swine fever virus, CSFV), *Pestivirus D* (Border disease virus, BDV), *Pestivirus E* (pronghorn pestivirus), *Pestivirus F* (Bungowannah virus), *Pestivirus G* (giraffe pestivirus), *Pestivirus H* (HoBi-like pestivirus, HoBiPeV), *Pestivirus I* (Aydin-like pestivirus), *Pestivirus J* (rat pestivirus), and *Pestivirus K* (atypical porcine pestivirus) (5).

*Pestivirus A, B*, and *H* species are infecting primarily cattle. To date, *Pestivirus A, B*, and *H* are classified into at least 21 (1a−1u), three (2a−2c), and four (a–d) (6, 7) subgenotypes, respectively.

The highest pestivirus prevalences were identified in cattle-producing countries where no control measures have been implemented, and their epidemiology in cattle is related to the pathogenetic mechanisms through which these viruses can cause both transient and persistent infections. Persistently infected (PI) animals, originating from a transient infection of pregnant cows or born from PI cows, shed large amounts of virus throughout their lives, thus ensuring viral persistence in the host population (2, 4).

In Italy, pestivirus infection has been reported in cattle all around the country since 1960 (8), with an increase of BVDV seroprevalence among dairy herds in the following years (9). Bovine viral diarrhea (BVD) was recognized as a relevant disease in Italian cattle herds from the beginning of 1990, as reported by regional studies on the disease and few local voluntary control programs (10, 11), and several BVDV vaccines were also available with an increase of commercialized vaccines for both beef and dairy cattle. Preliminary investigations showed a wide genetic heterogeneity among pestivirus strains circulating in cattle (12–14). To date, an eradication program has been successfully applied in Bolzano province, bordering Austria. In this area, dairy herds are prevalent and the program is based on tissue tag testing to directly detect PI newborn calves without using vaccination; a PI prevalence ≤0.01% has been reached so far. A compulsory program is also ongoing in Trentino province, whereas voluntary control programs are applied in few other northern regions (Piedmont, Veneto, Friuli-Venezia Giulia).

The aim of this review is to report an up-to-date overview about the genetic diversity of pestiviruses circulating in Italian cattle herds.

### Pestivirus A

BVDV-1 is the most prevalent pestivirus species in cattle population in Italy, reaching a percentage equal or higher than 96.9% of the detected strains according to the available data (7, 15). Several genotyping studies were carried out to characterize the pestivirus strains circulating in Italian cattle population (6, 12, 13, 15–24).

Recently, evidence of fourth subgenotypes, namely, BVDV-1r, BVDV-1s, BVDV-1t (6), and BVDV-1u (22), has increased the number of circulating subtypes previously reported (15), accounting for 15 out of 21 BVDV-1 subgenotypes recognized worldwide (7), circulating in Italy. A conflict of designation for BVDV subgenotypes has been reported, since indeed identical letter codes have been used for different BVDV-1 subgenotypes, namely, BVDV-1l and 1r, which were first described in two countries at close intervals (7).

The probability of detection of sporadic and low prevalent subgenotypes was likely increased due to the analysis of extensive collections of BVDV isolates; nevertheless, it has to be noticed that multiple BVDV-1 subgenotypes in cattle have been detected since the preliminary studies on a small sample size (12, 16) were carried out on 26 and 38 isolates, respectively. On the whole, four frequency and distribution patterns of BVDV-1 subgenotypes were identified in Italy (15) and updated by additional genotyping studies (6, 20, 23, 24): (1) high prevalent subgenotypes with a wide temporal–spatial distribution (BVDV-1b and 1e); (2) low prevalent subgenotypes with a widespread geographic distribution (BVDV-1a, 1d, 1h, and 1k); (3) low prevalent subgenotypes in restricted geographic areas (BVDV-1f); and (4) sporadic subgenotypes detected in few herds (≤5) (BVDV-1c, 1g 1j, 1l, 1r, 1s, 1t, 1u) in restricted areas (Table 1).

**Table 1:** Frequency of BVDV-1 subgenotypes in cattle.

**Subgenotype**	**Sequence no**.	**Years**	Geographic origin^*^	**References**
BVDV-1a	30	2000–2014	NCSI	(6, 15)
BVDV-1b	245	1995–2016	NCSI	(6, 15, 22–24)
BVDV-1c	3	2008–2010	CS	(6, 15)
BVDV-1d	28	1995–2010	NCS	(6, 15, 23, 24)
BVDV-1e	144	1996–2013	NCSI	(6, 15, 23, 24)
BVDV-1f	55	1999–2014	NC	(6, 15, 20)
BVDV-1g	5	2002–2010	NS	(6, 15)
BVDV-1h	28	1996–2016	NCSI	(6, 15, 22–24)
BVDV-1j	1	1995	N	(15)
BVDV-1k	8	2001–2011	NCSI	(6, 15)
BVDV-1l	2	2007	C	(6, 15)
BVDV-1r	4	2010–2012	NS	(6, 23)
BVDV-1s	1	2008	C	(6)
BVDV-1t	1	2013	I	(6)
BVDV-1u	5	2009–2015	SI	(22, 23)

* *N, northern Italy; C, central Italy; S, southern Italy; I, Islands*.

The BVDV-1 subgenotypes circulating in Italy have been reported in other countries (7), with exception of BVDV-1r, 1s, and 1t which were first and sporadically detected only in Italy (6) and BVDV-1u (22) which has been identified so far exclusively in China in different ruminant species, including cattle, water buffalo, and yak (25).

The relationships between the genetic diversity and geographic distribution of the BVDV-1 subgenotypes were investigated through phylogenetic analysis that includes spatiotemporal information in the tree inference, namely, phylogeographic analysis, in order to reconstruct the origin and viral dispersal routes. The largest virus dispersion occurred between the middle 1990s and the early 2000s; northern Italy was estimated to be a significant source area to other parts of the country of the most subgenotypes that are widespread at national level, namely, BVDV-1a, 1b, 1e, 1d, and 1h (19, 24) and also BVDV-1f (20). Considering that northern Italy is the area with the largest cattle population as well as one of the main cattle importing areas from other European countries, a possible gravity-like dynamic of the infection, originating in larger animal populations then diffusing to smaller ones following patterns of national commercial flow, has been hypothesized (19). The most prevalent subgenotypes (BVDV-1b and 1e) showed a common viral dispersal pattern with a continuous BVDV-1b and 1e interspersion from multiple areas, including other European countries until the end of the last century and with no evidence of significant geographical structure, while local circulation was prevalent in recent years with significant regional clusters (24). Accordingly, southern areas of the country concurred mainly to a restricted geographical circulation of BVDV-1b and 1e, as demonstrated by significant local transmission networks, suggesting a local maintenance of BVDV infection (24).

Molecular epidemiology and evolutionary phylodynamics allowed reconstructing the spatiotemporal westward dispersal of BVDV-1f in northern Italy and its introduction in Aosta Valley from Piedmont. Moreover, the combined approach of traditional and molecular epidemiology showed that BVDV-1f in Aosta Valley can be controlled only by monitoring the introduction of cattle from the Piedmont region (20).

### Pestivirus B

BVDV-2 was first identified in the USA (26) and then detected in several countries (27–30). Contaminated fetal calf sera or other biological products likely contributed to BVDV-2 introduction into Europe (31), where it circulates at lower rates than BVDV-1 (7). In Italy, BVDV-2 has been reported both in cattle (12) and in small ruminants since the 1990s (32). Despite the early identification in our country, BVDV-2 showed a sporadic frequency in cattle (12, 15, 33), with BVDV-2a representing the most prevalent subgenotype in this country (21, 22) as well as at a global level (7). BVDV-2c strains have been recently detected in southern Italy in cattle and to a greater extent in small ruminants (22). It is noteworthy that this BVDV-2 subgenotype, which was responsible for a severe outbreak of BVDV-2c infection occurred in Germany and the Netherlands during 2012–2014 (34, 35), had been found to circulate in Italy since 2004 (22).

### Pestivirus H

This emerging pestivirus species was first detected in South America (36) and then reported in South America, Europe, and Asia (37). Viruses circulating in South America, Europe, Thailand, and China were found to be closely related, other Asian HoBiPeV strains are highly divergent, and at least four different subgenotypes have been identified so far (6). In Europe, HoBiPeV was first detected in cattle in southern Italy in 2010 (14), although retrospective analysis of archival samples dates back its circulation in this country to 2007 (38). In Italian cattle, the virus was responsible for respiratory distress (14, 39), abortion (40), birth of PI calves (41, 42), mucosal disease (43), and gastroenteric signs (42), with severe economic losses in infected herds (42). Subsequently, an extensive collection of Italian cattle pestiviruses was analyzed to assess the frequency of this emerging virus in Italy. HoBiPeV strains were not further detected in cattle neither in southern Italy, where the virus was first detected (22, 23), nor all around the country (15).

## Pestivirus Genetic Variability Within Herds

The high diversity of circulating pestiviral strains affects also the BVDV variability at the herd level. A unique genetic variant was detected in the majority of herds, but co-circulation of different genetic pestiviruses (species and subgenotypes) was also observed in both dairy and beef herds, based on analysis of different strains within a narrow collection period (≤3 months) (15). In addition, the genetic variability of 5′UTR of the same BVDV subgenotype circulating within herds has been observed. This finding could indicate the introduction of a different strain or the genetic evolution of a single circulating strain, consistent with the mean evolutionary rate estimated for this genomic region, which is 9.3 × 10^−3^ substitutions/site/year, with a credibility interval between 4.8 and 14.7 substitutions for 1,000 nucleotides (19).

## Bovine Pestiviruses Circulating in Non-Bovine Ruminants

BVDV-1 was detected in water buffalo (*Bubalus bubalis*) in southern Italy (18, 22, 44). At the genetic typing, the strains were characterized as of BVDV-1b subgenotype, and a role in the etiology of abortion (44) and persistent infection in adult animals (18) in this ruminant species was suggested.

Circulation of BVDV-1 and BVDV-2 in sheep flocks was reported in southern Italy (22, 32). BVDV genetic typing allowed detecting BVDV-1a and 1f in sheep in central Italy (18), as well as BVDV-1e and BVDV-2c in both sheep and goat flocks in southern regions (22).

BVDV detection in wild ruminants is sporadic in Europe and analogous to BDV (45, 46), most probably dependent on a domestic source (47, 48). High mortality outbreaks caused by BDV infections were reported in Pyrenean chamois (*Rupicapra pyrenaica*) (49, 50), and introduction from sheep into the wildlife has been suggested for this virus (51).

Recently, BVDV-1 has been reported in wild ruminants in Italian central Apennines (52), with subgenotypes 1a and 1c being detected in red deer (*Cervus elaphus*), roe deer (*Capreolus capreolus*), and Apennine chamois (*Rupicapra pyrenaica ornata*), and in roe deer and Apennine chamois, respectively. No bovine pestivirus has been detected in wild ruminants in Italian Alps so far, and accordingly serological investigations suggest that pestivirus circulation either is absent or occurs at low prevalence in roe deer and red deer (53–55). In Alpine chamois (*Rupicapra rupicapra rupicapra*), no seropositivities were detected for BVDV by the virus neutralization test (53), while seroprevalences of 18% (55) and 25.5% (54) were observed for pestiviruses by the ELISA test, with no differentiation between BVDV and BDV.

## Discussion

**Figure 1:**
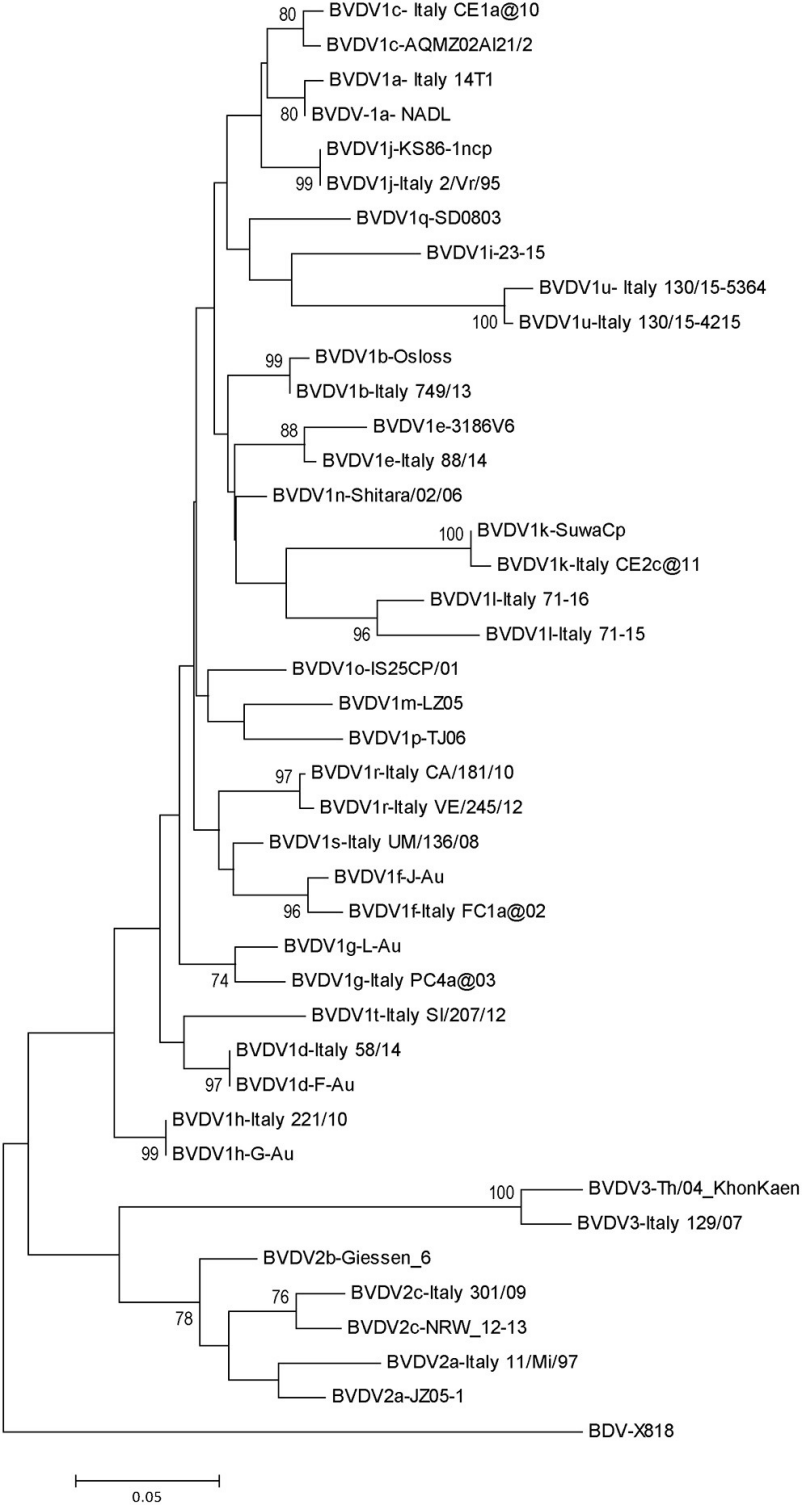
Phylogenetic tree based on the 5′-UTR of selected Italian sequences representative of pestiviruses detected in cattle and reference strains. Molecular evolutionary genetics analyses were performed with MEGA X using NJ method. Bootstrap values >70% are shown.

*Pestivirus A, B*, and *H* species have been identified in Italian cattle population with variable frequency and geographical distribution. Phylogenetic analysis of extensive collections of strains of the three bovine pestiviruses has allowed to detect several subgenotypes, accounting for 15 out of 21 BVDV-1 subgenotypes, two out three of BVDV-2, and one out of four HoBiPeV subgenotypes (Figure 1), recognized so far.

Based on the global distribution of BVDV subgenotypes recently reviewed (7) and integrated by other available literature (14, 22–24, 38), Italy is characterized by the highest genetic diversity of bovine pestiviruses among cattle-producing countries worldwide. Recently, a better understanding of national pestivirus distribution has been achieved, with the most prevalent subgenotypes being represented by BVDV-1b and 1-e (69.5%), having a wide distribution in all the country, including islands. Several subgenotypes (BVDV-1a, 1d, 1h, 1k) showed a wide dispersion despite the lower frequency compared to BVDV-1b and 1e. BVDV-1f is mainly restricted in northwestern Italy, namely, Piedmont and Aosta Valley, with evidence of the entry of BVDV-1f in Aosta Valley from Piedmont and transmission chains among local cattle farms (20). The remaining BVDV-1 subgenotypes were sporadically reported in Italy, but it has to be noticed that BVDV-1 heterogenicity is increasing due to the identification of novel subgenotypes (6) or emerging subgenotypes, such BVDV-1u (22), which had been previously reported exclusively in China (25).

BVDV-2 displays a very limited circulation in cattle (12, 33), whereas a higher frequency of detection has been observed in small ruminants in southern Italy (22, 32), where livestock breeding is mainly characterized by semi-intensive and extensive farming of sheep and goat flocks.

The sporadic frequency of HoBiPeV in Italy and the absence of circulation in other European countries support the hypothesis that HoBiPeV was introduced to southern Italian cattle herds through contaminated biological products, rather than infected animals (23, 36).

On the whole, the high level of BVDV-1 genetic heterogeneity and the spatial distribution of BVDV are mainly attributable to the cattle trade within the country and to introduction of viral strains from other countries, in the absence of any control measures. Northern Italy was estimated to be a source area to other parts of the country of subgenotypes that are now widespread at the national level (19, 24). In addition, biological products contaminated by fetal bovine serum have to be considered as possible source for introduction of bovine pestivirus species and subgenotypes into new areas (6, 31, 56).

A data integration of the cattle movement dataset with the pestivirus status is advisable to optimize the outcome of molecular characterization of pestiviruses, performing an accurate contact tracing among farms and investigating transmission pathways among different areas. Moreover, the genetic diversity of Italian pestiviral strains may have diagnostic and immunological implications, affecting the performance of diagnostic tools and the full cross-protection elicited by commercially available vaccines (57–59). In this respect, implementation and strengthening of coordinated approaches for bovine pestivirus control in Italy are recommended.

The current European situation of eradication and control programs for pestiviruses in cattle is rapidly evolving, with an increased number of countries applying systematic control measures at the national level (60, 61). For BVDV mitigation, it would be extremely important to regulate the cattle trade according to the disease status of a farm or a region and also to increase control and restriction of trade of biological products containing fetal bovine serum (4).

Dairy farms are recently identified as the key drivers of pestivirus persistence and dispersion in Italy, and control measures targeting these farms would lead significant reduction in the pestivirus circulation in Italian cattle to a higher extent than targeting other production compartments (62).

## Author Contributions

All authors listed have made a substantial, direct and intellectual contribution to the work, and approved it for publication.

## Conflict of Interest

The authors declare that the research was conducted in the absence of any commercial or financial relationships that could be construed as a potential conflict of interest.

## References

[1] Houe H. Economic impact of BVDV infection in dairies. Biologicals. (2003) 31:137–43. 10.1016/S1045-1056(03)00030-7 12770546

[2] Brock KV. The many faces of bovine viral diarrhea virus. Vet Clin North Am Food Anim Pract. (2004) 20:1–3. 10.1016/j.cvfa.2003.12.002 15062470

[3] Ridpath J. The contribution of infections with bovine viral diarrhea viruses to bovine respiratory disease. Vet Clin North Am Food Anim Pract. (2010) 26:335–48. 10.1016/j.cvfa.2010.04.003 20619188

[4] Evans CA Pinior B Larska M Graham D Schweizer M Guidarini C. Global knowledge gaps in the prevention and control of bovine viral diarrhoea (BVD) virus. Transbound Emerg Dis. (2019) 66:640–52. 10.1111/tbed.13068 30415496

[5] Smith DB Meyers G Bukh J Gould EA Monath T Muerhoff AS. Proposed revision to the taxonomy of the genus Pestivirus, family Flaviviridae. J Gen Virol. (2017) 98:2106–12. 10.1099/jgv.0.000873 28786787 PMC5656787

[6] Giammarioli M Ceglie L Rossi E Bazzucchi M Casciari C Petrini S. Increased genetic diversity of BVDV-1: recent findings and implications thereof. Virus Genes. (2015) 50:147–51. 10.1007/s11262-014-1132-2 25349062

[7] Yeşilbag K Alpay G Becher P. Variability and global distribution of subgenotypes of bovine viral diarrhea virus. Viruses. (2017) 9:128. 10.3390/v9060128 28587150 PMC5490805

[8] Ademollo A Battelli C. Viral diarrhea-mucosal disease" complex in cattle in Italy. Bull Off Int Epizoot. (1966) 66:421–31. 6011905

[9] Cavirani S Luini M Allegri G Fabbri M Bottarelli E Flamini CF. Un decennio di ricerche sierologiche sulla diffusione di bovine herpesvirus 1 (BHV-1), bovine viral diarrhea Virus (BVDV) e bovid herpesvirus 4 (BHV-4). Sel Vet. (1992) 33:459–67.

[10] Ferrari G Scicluna MT Bonvicini D Gobbi C Della V.erità F, Valentini A, et al. Bovine virus diarrhoea (BVD) control programme in an area in the Rome province (Italy). Vet Microbiol. (1999) 64:237–45. 10.1016/S0378-1135(98)00273-9 10028176

[11] Luzzago C Frigerio M Zecconi A. BVD control program in Lecco and Como provinces (Italy): herd risk categories to modulate interventions. In: Proceedings of the 2nd European Symposium on BVDV Control. Oporto (2004). p. 100.

[12] Luzzago C Bandi C Bronzo V Ruffo G Zecconi A. Distribution pattern of bovine viral diarrhoea virus strains in intensive cattle herds in Italy. Vet Microbiol. (2001) 83:265–74. 10.1016/S0378-1135(01)00429-1 11574174

[13] Vilček Š Paton DJ Durkovic B Strojny L Ibata G Moussa A. Bovine viral diarrhoea virus genotype 1 can be separated into at least eleven genetic groups. Arch Virol. (2001) 146:99–115. 10.1007/s007050170194 11266221

[14] Decaro N Lucente MS Mari V Cirone F Cordioli P Camero M. atypical pestivirus and severe respiratory disease in calves, Europe. Emerg Infect Dis. (2011) 17:1549–52. 10.3201/eid1708.101447 21801648 PMC3381567

[15] Luzzago C Lauzi S Ebranati E Giammarioli M Moreno A Cannella V. Extended genetic diversity of bovine viral diarrhea virus and frequency of genotypes and subtypes in cattle in Italy between 1995 and 2013. Biomed Res Int. (2014) 2014:147145. 10.1155/2014/147145 25045658 PMC4090534

[16] Falcone E Cordioli P Tarantino M Muscillo M La Rosa G Tollis M. Genetic heterogeneity of bovine viral diarrhoea virus in Italy. Vet Res Commun. (2003) 27:485–94. 10.1023/A:1025793708771 14582747

[17] Ciulli S Galletti E Battilani M Scagliarini A Gentile A Morganti L. Genetic typing of bovine viral diarrhoea virus: evidence of an increasing number of variants in Italy. New Microbiol. (2008) 31:263–71. 18623993

[18] Giammarioli M Pellegrini C Casciari C Rossi E De Mia GM. Genetic diversity of bovine viral diarrhea virus 1: Italian isolates clustered in at least seven subgenotypes. J Vet Diagn Invest. (2008) 20:783–8. 10.1177/104063870802000611 18987229

[19] Luzzago C Ebranati E Sassera D Lo Presti A Lauzi S Gabanelli E. Spatial and temporal reconstruction of bovine viral diarrhea virus genotype 1 dispersion in Italy. Infect Genet Evol. (2012) 12:324–31. 10.1016/j.meegid.2011.12.007 22210133

[20] Cerutti F Luzzago C Lauzi S Ebranati E Caruso C Masoero L. Phylogeography, phylodynamics and transmission chains of bovine viral diarrhea virus subtype 1f in Northern Italy. Infect Genet Evol. (2016) 45:262–7. 10.1016/j.meegid.2016.09.007 27619057

[21] Bazzucchi M Bertolotti L Ceglie L Giammarioli M Rossi E Rosati S. Complete nucleotide sequence of a novel bovine viral diarrhea virus subtype 1 isolate from Italy. Arch Virol. (2017) 162:3545–8. 10.1007/s00705-017-3486-y 28717858

[22] Decaro N Lucente MS Lanave G Gargano P Larocca V Losurdo. Evidence for circulation of bovine viral diarrhoea virus type 2c in ruminants in Southern Italy. Transbound Emerg Dis. (2017) 64:1935–44. 10.1111/tbed.12592 27878974

[23] Lanave G Decaro N Lucente MS Guercio A Cavaliere N Purpari G. Circulation of multiple subtypes of bovine viral diarrhoea virus type 1 with no evidence for HoBi-like pestivirus in cattle herds of southern Italy. Infect Genet Evol. (2017) 50:1–6. 10.1016/j.meegid.2017.02.009 28189886

[24] Ebranati E Lauzi S Cerutti F Caruso C Masoero L Moreno A. Highlighting priority areas for bovine viral diarrhea control in Italy: a phylogeographic approach. Infect Genet Evol. (2018) 58:258–68. 10.1016/j.meegid.2018.01.006 29329686

[25] Deng M Ji S Fei W Raza S He C Chen Y. Prevalence study and genetic typing of bovine viral diarrhea virus (bvdv) in four bovine species in China. PLoS ONE. (2015) 10:e0121718. 10.1371/journal.pone.0121718 25849315 PMC4388703

[26] Pellerin C van den Hurk J Lecomte J Tijssen P. Identification of a new group of bovine viral diarrhea virus strains associated with severe outbreaks and high mortalities. Virology. (1994) 203:260–8. 10.1006/viro.1994.1483 8053150

[27] Nagai M Sato M Nagano H Pang H Kong X Murakami T. Nucleotide sequence homology to bovine viral diarrhea virus 2 (BVDV 2) in the 5′ untranslated region of BVDVs from cattle with mucosal disease or persistent infection in Japan. Vet Microbiol. (1998) 60:271–6. 10.1016/S0378-1135(98)00158-8 9646457

[28] Letellier C Kerkhofs P Wellemans G Vanopdenbosch E. Detection and genotyping of bovine diarrhea virus by reverse transcription-polymerase chain amplification of the 5′ untranslated region. Vet Microbiol. (1999) 64:155–67. 10.1016/S0378-1135(98)00267-3 10028170 PMC7117503

[29] Tajima M Frey HR Yamato O Maede Y Moennig V Scholz H. Prevalence of genotypes 1 and 2 of bovine viral diarrhea virus in Lower Saxony, Germany. Virus Res. (2001) 76:31–42. 10.1016/S0168-1702(01)00244-1 11376844

[30] Jackova A Novackova M Pelletier C Audeval C Gueneau E Haffar A. The extended genetic diversity of BVDV-1: typing of BVDV isolates from France. Vet Res Commun. (2008) 32:7–11. 10.1007/s11259-007-9012-z 17657577

[31] Falcone E Tollis M Conti G. Bovine viral diarrhea disease associated with a contaminated vaccine. Vaccine. (1999) 18:387–8. 10636817 10.1016/s0264-410x(99)00244-3

[32] Pratelli A Martella V Cirone F Buonavoglia D Elia G Tempesta M. Genomic characterization of pestiviruses isolated from lambs and kids in southern Italy. J Virol Methods. (2001) 94:81–5. 10.1016/S0166-0934(01)00277-4 11337042

[33] Decaro N Camero M Elia G Martella V Pratelli A Gargano P. Malattia delle mucose da BVDV tipo 2: descrizione di un focolaio in Puglia. Large Anim Rev. (2004) 10:29–34.

[34] Gethmann J Homeier T Holsteg M Schirrmeier H Saßerath M Hoffmann. BVD-2 outbreak leads to high losses in cattle farms in Western Germany. Heliyon. (2015) 1:e00019. 10.1016/j.heliyon.2015.e00019 27441213 PMC4939757

[35] Wernike K Schirrmeier H Strebelow HG Beer M. Eradication of bovine viral diarrhea virus in Germany-Diversity of subtypes and detection of live-vaccine viruses. Vet Microbiol. (2017) 208:25–9. 10.1016/j.vetmic.2017.07.009 28888645

[36] Bauermann FV Ridpath JF Weiblen R Flores EF. HoBi-like viruses: an emerging group of pestiviruses. J Vet Diagn Invest. (2013) 25:6–15. 10.1177/1040638712473103 23345268

[37] Decaro N. HoBi-like pestivirus and reproductive disorders. Front Vet Sci. (2020) 7:622447. 10.3389/fvets.2020.622447 33415134 PMC7782308

[38] Decaro N Mari V Lucente MS Sciarretta R Elia G Ridpath. Detection of a Hobi-like virus in archival samples suggests circulation of this emerging pestivirus species in Europe prior to 2007. Vet Microbiol. (2013) 167:307–13. 10.1016/j.vetmic.2013.09.006 24095625

[39] Decaro N Mari V Pinto P Lucente MS Sciarretta R Cirone F. Hobi-like pestivirus: both biotypes isolated from a diseased animal. J Gen Virol. (2012) 93:1976–83. 10.1099/vir.0.044552-0 22764319

[40] Decaro N Lucente MS Mari V Sciarretta R Pinto P Buonavoglia D. Hobi-like pestivirus in aborted bovine fetuses. J Clin Microbiol. (2012) 50:509–12. 10.1128/JCM.05887-11 22162547 PMC3264192

[41] Decaro N Losurdo M Lucente MS Sciarretta R Mari V Larocca V. Persistent infection caused by Hobi-like pestivirus. J Clin Microbiol. (2013) 51:1241–3. 10.1128/JCM.03134-12 23325822 PMC3666814

[42] Decaro N Lucente MS Losurdo M Larocca V Elia G Occhiogrosso L. HoBi-like pestivirus and its impact on cattle productivity. Transbound Emerg Dis. (2016) 63:469–73. 10.1111/tbed.12529 27390140

[43] Decaro N Lanave G Lucente MS Mari V Varello K Losurdo M. Mucosal disease-like syndrome in a calf persistently infected by Hobi-like pestivirus. J Clin Microbiol. (2014) 52:2946–54. 10.1128/JCM.00986-14 24899039 PMC4136150

[44] Martucciello A De Mia GM Giammarioli M De Donato I Iovane G Galiero G. Detection of Bovine viral diarrhea virus from three water buffalo fetuses (*Bubalus bubalis*) in Southern Italy. J Vet Diagn Invest. (2009) 21:137–40. 10.1177/104063870902100123 19139516

[45] Martin C Duquesne V Adam G Belleau E Gauthier D Champion JL. Pestiviruses infections at the wild and domestic ruminants interface in the French Southern Alps. Vet Microbiol. (2015) 175:341–8. 10.1016/j.vetmic.2014.11.025 25532780

[46] Cerutti F Caruso C Modesto P Orusa R Masoero L Acutis PL. The genome of Border disease virus genotype 8 from chamois bynext generation sequencing. Vet Ital. (2019) 55:103–5. 10.12834/VetIt.1768.9338.1 30794320

[47] Casaubon J Vogt HR Stalder H Hug C Ryser-Degiorgis MP. Bovine viral diarrhea virus in free-ranging wild ruminants in Switzerland: low prevalence of infection despite regular interactions with domestic livestock. BMC Veterinary Research. (2012) 8:204. 10.1186/1746-6148-8-204 23107231 PMC3514304

[48] Rodríguez-Prieto V Kukielka D Rivera-Arroyo B Martínez-López B de las Heras AI Sánchez-Vizcaíno JM. Evidence of shared bovine viral diarrhea infections between red deer and extensively raised cattle in south-central Spain. BMC Vet Res. (2016) 12:11. 10.1186/s12917-015-0630-3 26767363 PMC4712561

[49] Marco I Rosell R Cabezón O Mentaberre G Casas E Velarde R. Border disease virus among Chamois, Spain. Emerg Infect Dis. (2009) 15:448–51. 10.3201/eid1503.081155 19239761 PMC2681124

[50] Fernández-Sirera L Cabezón O Allepuz A Rosell R Riquelme C Serrano E. Two different epidemiological scenarios of border disease in the populations of pyrenean chamois (Rupicapra p. *pyrenaica)* after the first disease outbreaks. PLoS ONE. (2012) 7:e51031. 10.1371/journal.pone.0051031 23251417 PMC3519488

[51] Luzzago C Ebranati E Cabezón O Fernández-Sirera L Lavín S Rosell R. Spatial and temporal phylogeny of border disease virus in Pyrenean Chamois (*Rupicapra p. pyrenaica)*. PLoS ONE. (2016) 11:e0168232. 10.1371/journal.pone.0168232 28033381 PMC5199066

[52] Ricci S Bartolini S Morandi F Cuteri V Preziuso S. Genotyping of Pestivirus A (Bovine Viral Diarrhea Virus 1) detected in faeces and in other specimens of domestic and wild ruminants at the wildlife-livestock interface. Vet Microbiol. (2019) 235:180–7. 10.1016/j.vetmic.2019.07.002 31383300

[53] Citterio CV Luzzago C Sala M Sironi G Gatti P Gaffuri A. Serological study of a population of alpine chamois (Rupicapra r rupicapra) affected by an outbreak of respiratory disease. Vet Rec. (2003) 153:592–6. 10.1136/vr.153.19.592 14640327

[54] Olde Riekerink RGM Dominici A Barkema HW de Smit AJ. Seroprevalence of pestivirus in four species of alpine wild ungulates in the High Valley of Susa, Italy. Vet Microbiol. (2005) 108:297–303. 10.1016/j.vetmic.2005.04.014 15922522

[55] Gaffuri A Giacometti M Tranquillo VM Magnino S Cordioli P Lanfranchi P. Serosurvey of roe deer, chamois and domestic sheep in the Central Italian Alps. J Wildl Dis. (2006) 42:685–90. 10.7589/0090-3558-42.3.685 17092903

[56] Pecora A Perez Aguirreburualde MS Ridpath JF Dus Santos MJ. molecular characterization of pestiviruses in fetal bovine sera originating from Argentina: evidence of circulation of HoBi-like viruses. Front Vet Sci. (2019) 6:359. 10.3389/fvets.2019.00359 31681812 PMC6805694

[57] Bauermann FV Flores EF Ridpath JF. Antigenic relationships between Bovine viral diarrhea virus 1 and 2 and HoBi virus: possible impacts on diagnosis and control. J Vet Diagn Invest. (2012) 24:253–61. 10.1177/1040638711435144 22379042

[58] Decaro N Mari V Sciarretta R Lucente MS Camero M Losurdo M. Comparison of the cross-antibody response induced in sheep by inactivated bovine viral diarrhoea virus 1 and Hobi-like pestivirus. Res Vet Sci. (2013) 94:806–8. 10.1016/j.rvsc.2012.11.016 23261155

[59] Mari V Losurdo M Lucente MS Lorusso E Elia G Martella V. Multiplex real-time RT-PCR assay for bovine viral diarrhea virus type 1, type 2 and HoBi-like pestivirus. J Virol Methods. (2016) 229:1–7. 10.1016/j.jviromet.2015.12.003 26709100 PMC7113868

[60] Ståhl K Alenius S. BVDV control and eradication in Europe–an update. Jpn J Vet Res. (2012) 60:S31–39. 22458198

[61] Moennig V Becher P. Control of bovine viral diarrhea. Pathogens. (2018) 7:29. 10.3390/pathogens7010029 PMC5874755 29518049

[62] Iotti B Valdano E Savini L Candeloro L Giovannini A Rosati S. Farm productive contexts and the dynamics of bovine viral diarrhea (BVD) transmission. Prev Vet Med. (2019) 165:23–33. 10.1016/j.prevetmed.2019.02.001 30851924

